# Exploring the Metabolic Heterogeneity of Cancers: A Benchmark Study of Context-Specific Models

**DOI:** 10.3390/jpm11060496

**Published:** 2021-06-01

**Authors:** Mahdi Jalili, Martin Scharm, Olaf Wolkenhauer, Mehdi Damaghi, Ali Salehzadeh-Yazdi

**Affiliations:** 1 Hematology, Oncology and SCT Research Center, Tehran University of Medical Sciences, Tehran 14114, Iran; 2 Department of Systems Biology and Bioinformatics, University of Rostock, 18051 Rostock, Germany; martin.scharm@uni-rostock.de (M.S.); olaf.wolkenhauer@uni-rostock.de (O.W.); 3 Wallenberg Research Centre, Stellenbosch Institute for Advanced Study (STIAS), Stellenbosch University, 10 Marais Street, Stellenbosch 7600, South Africa; 4 Department of Cancer Physiology, Moffitt Cancer Center and Research Institute, Tampa, FL 33612, USA; mehdi.damaghi@moffitt.org; 5 Department of Oncologic Sciences, Morsani College of Medicine, University of South Florida, Tampa, FL 33612, USA

**Keywords:** genome-scale metabolic model, data integration, Warburg effect, metabolic pattern, FBA-based feature, cancer metabolism

## Abstract

Metabolic heterogeneity is a hallmark of cancer and can distinguish a normal phenotype from a cancer phenotype. In the systems biology domain, context-specific models facilitate extracting physiologically relevant information from high-quality data. Here, to utilize the heterogeneity of metabolic patterns to discover biomarkers of all cancers, we benchmarked thousands of context-specific models using well-established algorithms for the integration of omics data into the generic human metabolic model Recon3D. By analyzing the active reactions capable of carrying flux and their magnitude through flux balance analysis, we proved that the metabolic pattern of each cancer is unique and could act as a cancer metabolic fingerprint. Subsequently, we searched for proper feature selection methods to cluster the flux states characterizing each cancer. We employed PCA-based dimensionality reduction and a random forest learning algorithm to reveal reactions containing the most relevant information in order to effectively identify the most influential fluxes. Conclusively, we discovered different pathways that are probably the main sources for metabolic heterogeneity in cancers. We designed the GEMbench website to interactively present the data, methods, and analysis results.

## 1. Introduction

**Figure 1:**
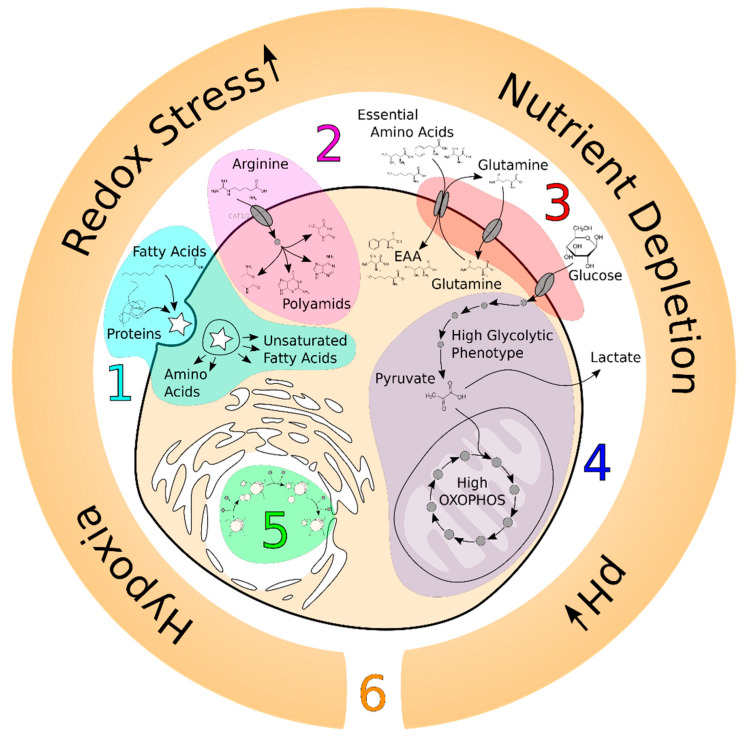
Metabolic hallmarks of cancer. Here, we depict six main metabolic hallmarks of cancer; (1) use of opportunistic modes of nutrient acquisition, (2) high demand for nitrogen, (3) deregulated uptake of glucose and essential amino acids, (4) use of high glycolysis/tricarboxylic acid (TCA) cycle intermediates for biosynthesis, (5) alterations in metabolite-driven gene regulation, and (6) microenvironment.

The cornerstone of metabolic research in oncology was laid by Otto Warburg in the 1920s when he discovered the main metabolic hallmark of cancer, aerobic glycolysis [1]. Aerobic glycolysis (the Warburg effect) refers to a common feature of cancer cell metabolism (although this is not a universal feature of proliferating cancer cells [2]) in which cancer cells take up glucose at high rates and break it down into lactate in the presence or absence of oxygen. Since then, a large quantity of high-throughput data have unveiled other metabolic hallmarks of cancer (Figure 1) [3], however, the metabolic patterns of different cancers are still the subject of inquiry [4]. A specific metabolic pattern can be defined as a downstream informative layer (rather than upstream data and transcriptome and proteome data) of a biological system [5] that is experimentally obtained by metabolomics analyses or theoretically by flux balance analyses (FBAs). Various experimental studies showed that studying the heterogeneity in the metabolic pattern of cancers contributes to better stratification of the disease and its treatment, which can also be correlated with drug sensitivity [6,7]. However, the metabolic patterns of cancers could not be fully elucidated by recent metabolomics studies due to complexity and limitations of the techniques and analysis [8].

Aided by metabolic modeling approaches, in particular a genome-scale metabolic model (GEM), studies on cancer cell metabolism have expanded our understanding of the underlying mechanisms of cancer development as well as the identification of diagnostic biomarkers, potential drug targets, and prognostic biomarkers [9]. The GEM can also be tailored to generate the context-specific GEM, using omics data integration approaches [10]. These context-specific models facilitate the unveiling of the genotype–phenotype relationship by calculating the flux states and extracting physiologically relevant information from high-quality data [11].

Omics data integration approaches are often developed to build cancer-specific GEMs (CGEMs) (Table 1). These types of studies highlight the importance of understanding the evolution of cancer metabolism and metabolic phenotypes [9] to discover cancers’ vulnerabilities, leading to novel prognostic biomarkers and therapeutics [12]. Increasing interest in GEM and CGEM approaches has induced a line of new publications focusing on the benchmarking of the tools used to generate context-specific models. All agree that none of these algorithms systematically outperforms the others and each has specific pros and cons depending on the type of data availability to tailor the GEM [13,14,15].

Up to now, there has been no comprehensive benchmark for the assessment of the metabolic phenotype patterns as biomarkers of all cancers (the active reactions carrying fluxes and their flux magnitude, FBA-based feature) and cancer cells. To understand the influence of context-specific extraction methods on the metabolic pattern and how likely a certain metabolic pattern leads to specific metabolic hallmarks of cancer, we generated 3740 CGEMs. In addition, we benchmarked the integration of all types of publicly available expression omics data into Recon3D by four algorithms to (i) evaluate the quality of a context-specific model of cancer, (ii) evaluate the impact of omics data and integration algorithms on the behavior of a CGEM, (iii) assess the metabolic patterns of different cancer types and (iv) suggest the main features of heterogeneity in cancer metabolism. Accordingly, we integrated omics data from both human cancer cell lines and actual patients’ samples (microarray gene expression, RNA-seq transcriptome, and proteomics data) into Recon3D by GIMME, iMAT, INIT, and FASTCORE algorithms. We then employed machine learning (ML) algorithms to both leverage the discrepancies in metabolic flux states of CGEMs (FBA-based feature) and also to determine the main metabolic features allowing a classification of various cancers. The results indicate that the active reactions and their corresponding flux magnitude for each CGEM are unique to each cancer type that can act as their metabolic fingerprint. We also discovered that extracellular transport, peptide metabolism, fatty acid synthesis, vitamin A metabolism, mitochondrial transporters, and the pentose phosphate pathway are probably the main varied pathways in all cancers. Therefore, they are the main sources of metabolic heterogeneity in different cancers. This discovery sheds more light on the cancer metabolism domain and can delineate potential future scenarios for personalized medicine.

## 2. Materials and Methods

### 2.1. Data Collection and Preprocessing

Data were extracted from human cancer-specific omics data from two different sources: (i) human cancer cell lines and (ii) patients’ samples. Each data source included three experimental platforms: microarray gene expression, RNA-seq gene sequencing, and proteomics data. Table 2 shows the summary of sources, platforms, and the number of samples we used.

#### 2.1.1. Human Cancer Cell Line Data

##### Human Protein Atlas (HPA)

The HPA is a freely available database including the Tissue Atlas, Cell Atlas, and Pathology Atlas. The Cell Atlas contains mRNA expression profiles of 64 cell lines from RNA sequencing. After filtering the non-metastatic human cancer cell lines, 32 cell lines were downloaded [24].

##### EMTAB-37

ArrayExpress is an archive of functional genomics data that stores high-throughput functional genomics data and provides them for reuse to the research community. The transcriptomics profiles of 317 various cancer cell lines were downloaded from (https://www.ebi.ac.uk/arrayexpress/experiments/E-MTAB-37/samples/) (accessed on 23 September 2020) [25].

##### NCI-60 Proteome

ProteomicsDB is an effort of the Technische Universität München that is dedicated to expedite the identification of various proteomes and their use across the scientific community. We used the results of the Global Proteome Analysis study. Gholami et al. employed an MS-based proteomics analysis and provided quantitative proteome profiles of all 59 cell lines of the NCI-60 panel. The dataset contains 10,350 identified proteins [26].

#### 2.1.2. Human Cancer Patient Data

##### TCGA

The Cancer Genome Atlas (TCGA) is a joint effort between the National Cancer Institute and the National Human Genome Research Institute that generated high-throughput data. The RNA-seq data were obtained using the GDC Data Portal platform. In total, 11,571 files for 10,672 cases were found. The metadata file was downloaded and only files of primary tumor- or primary blood-derived cancer bone marrow or peripheral were selected and, finally, 10,322 files were downloaded. After the combination of the same disease types, 202 different cancer types were prepared for the current study [27].

##### GSE2109

The Gene Expression Omnibus (GEO) is a public functional genomics data repository that stores microarray- and sequence-based data [28]. The results of DNA microarray analysis of “The Expression Project for Oncology” (expO) from the International Genomics Consortium were downloaded from (https://www.ncbi.nlm.nih.gov/geo/query/acc.cgi?acc=GSE2109) (accessed on 23 September 2020). Among 2158 patient samples, the metastatic and ambiguous samples were removed and 1895 samples from 315 different cancer types were selected. In total, 24,442 unique genes exist in this dataset [28].

##### ProteomeXchange (PX)

The ProteomeXchange (PX) Consortium of proteomics resources (http://www.proteomexchange.org) (accessed on 23 September 2020) provides a standard portal for submission and dissemination of mass spectrometry proteomics data [29]. We extracted 10 datasets of four different cancer types containing 11,961 identified proteins.

##### Data Preprocessing

All replicated data were averaged and normalized between 0 and 1 (min–max normalization) using a z-score scaling formula,

$$\frac{Value-min}{max-min}$$

### 2.2. Genome-Scale Metabolic Models (GEMs)

Recon3D [30] version 3.01 was downloaded from the VMH database [31]. Recon3D is a manually curated GEM which contains 13,543 reactions, 4140 metabolites, and 3697 genes. All exchange reactions used the original flux bounds of the Reconn3D model. The original model was converted to a flux consistent model (i.e., all reactions are able to carry fluxes) using the ‘findFluxConsistentSubset’ function of COBRA Toolbox and the ‘fastcc’ algorithm of ‘fastcore’ with a reaction activity threshold of 1^−10^ [32]. The final consistent model, used in the study, contained 3256 metabolites and 11,961 reactions. For all constructed context-specific models, six reactions were reserved and prevented from deletion, ‘biomass_reaction’, ‘DM_atp_c_’, ‘GAPD’, ‘ATPS4mi’, ‘D_LACt2’, and ‘O2t’.

#### Data Processing and Extraction of Context-Specific Model

The gene/protein expression data were mapped to model reactions using gene–protein–reaction (GPR) rules. For genes with ‘AND’ logic (enzyme complex), the minimum expression, and for ‘OR’ logic (isozyme), the maximum expression of the corresponding genes, were used. Reactions were not assigned a value if GPR or expression data were missing. Then, we used four prominent integration algorithms to generate cancer-specific GEMs (CGEM). The integration algorithms can be classified in many different ways on the basis of the underlying methodology. In 2015, Estévez and Nikoloski [33] stratified these methods into three groups: 1. GIMME- and GIM3E-like methods. This group refers to two-step approaches with a metabolic functionality (objective function) being optimized via FBA. Then, the obtained optimal value is employed to minimize the discrepancies between fluxes and data; 2. iMAT- and INIT-like methods. This group of algorithms determines the binary reaction status (i.e., active or inactive) which are in good agreement with the associated data state; and 3. MBA-, mCADRE-, and fastCORE-like methods. This group defines a core set of active reactions and then finds the minimum essential set of reactions to satisfy the consistency condition of the model. In this study, we selected four algorithms, GIMME, INIT, iMAT, and FASTCORE. The created CGEMs were optimized for maximization of biomass reaction [34] using the ‘optimizeCbModel’ function of COBRA Toolbox, and reaction flux values were selected. 

### 2.3. Validation of CGEMs by Two Known Hallmarks of Cancer

#### 2.3.1. Metabolic Hallmark of CGEMs (Warburg Score)

The quantification of the Warburg effect in CGEMs was formulated in two studies [20,35]. The glycolytic to oxidative ATP flux ratio (AFR or BEC) and the ratio of the glycolytic versus oxidative capacity (EOR) as two indices of the metabolic hallmarks of cancer for CGEMs were defined. We calculated these two scores by the following formulations for all CGEMs:

$$\mathrm{AFR}\;\mathrm{score}\;=\frac{Flux_{GAPD}}{Flux_{ATPS4mi}}$$

$$\mathrm{EOR}\;\mathrm{score}=\frac{Flux_{D\_LACt2}}{Flux_{O2t}}$$

#### 2.3.2. The Gene Set Hallmark of Cancer

Liberzon et al. [36] presented the gene set hallmark of cancer containing 197 genes [37]. Here, we calculated the total number of cancer hallmark genes presented in all CGEMs.

### 2.4. Characterization of Metabolic Pattern of CGEMs

#### 2.4.1. Jaccard Index

The Jaccard similarity coefficient [38,39] was used as a similarity index to calculate the proportion of overlap between two sets of features (the number of active reactions in CGEMs). The Jaccard index is the proportion of shared elements between set A and set B relative to the total number of the union of the elements. It is defined below:

$${\mathrm{Jaccard}\;\mathrm{index}}_{i,j}=\frac{\left|N_{Ri}\cap N_{Rj}\right|}{\left|N_{Ri}\cup N_{Rj}\right|}$$

where $N_{Ri}$ are active reactions of disease *i* and $N_{Rj}$ are active reactions of disease *j*. The range is between 0 and 1.

Indeed, active reactions were defined as the reactions which remained in the constructed context-specific model after applying the integration method into the original model (consistent Recon3D). For each disease pair, the number of remaining reactions in the model’s subsystems (109 different subsystems) was determined. The Jaccard index for each subsystem was calculated separately and the final Jaccard index was indicated by the mean of these indices.

#### 2.4.2. Bland–Altman Plot

The Bland–Altman plot and limits of agreement (LoAs) is a standard method for the assessment of the results of the two methods [40]. The difference between the two methods is plotted against the averages of the two methods and the LoAs are defined as the mean difference ±1.96 times the standard deviation of the differences. The plots were interpreted informally and if the differences are not clinically important, the two methods may be used interchangeably.

#### 2.4.3. Principal Component Analysis (PCA)

Principal component analysis is used to extract the important information from multidimensional correlated variables and to provide a set of new uncorrelated variables called principal components. The goal of PCA is to identify directions showing the highest variations in the data and, thus, carrying the main information. The first two principal components have the largest possible variance or data variability and can be visualized graphically. The flux distribution data were classified by the ‘prcomp’ function of the stats package in R and results visualized by the ‘factoextra’ R package [41].

#### 2.4.4. Machine Learning Method (Random Forest)

Random forests are ensemble classifiers that aggregate the results of many individual decision trees. We used the ‘randomForest’ R package [42] with two hyperparameters: the number of training trees (nTree) and the number of predictors to consider at each split point (mTry). The default settings of nTree = 500 and mTry as the square root of the number of predictor variables were used in this study. The PCA results were used as the response variable in the random forest (RF) analysis and only flux distributions which had two or more classes in PCA were included in the analysis. All flux data were used as a training set. The results of RF were interpreted and visualized by the ‘randomForestExplainer’ R package [43].

### 2.5. Implementation

All codes were implemented in MATLAB and R. COBRA Toolbox 2.0 [44] in MATLAB 2018b and IBM CPLEX as a solver were used. The website for GEMbench at https://gembench.bio.informatik.uni-rostock.de/ (accessed on 23 September 2020) is basic HTML with plain CSS layout. User interaction with the website was realized using JavaScript. The figures were created in D3.js (https://d3js.org/) (accessed on 23 September 2020).

## 3. Results

### 3.1. Construction of Cancer-Specific GEMs (CGEMs)

**Figure 2:**
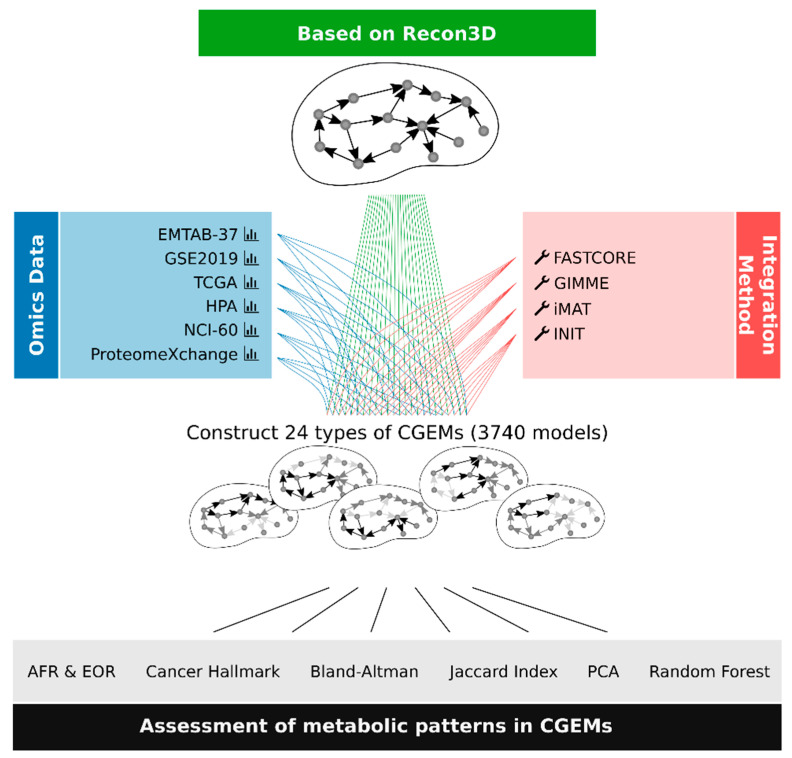
The workflow of the six different integrated human cancer omics datasets into GEM using four integration approaches. We generated 24 types of dataset–algorithm CGEMs. Afterwards, we evaluated the CGEMs by three indices (AFR, EOR, and cancer gene set). Using FBA, we calculated metabolic flux states for all CGEMs and then utilized different statistical and machine learning approaches to find the main metabolic features of CGEMs.

Cancer-specific GEMs are the de facto standard to analyze cancer behaviors. To comprehensively assess the metabolic patterns of different cancer types, we integrated six different human cancer omics datasets that are publicly available (Table 2) into the generic human GEM (Recon3D) using four integration approaches (GIMME, iMAT, INIT, and FASTCORE). The six omics datasets are composed of (i) human cancer cell line data including 32 RNA-seq profiles (HPA database), 317 microarray profiles (EMTAB-37 dataset) and 59 proteome profiles (NCI-60 proteome dataset), (ii) human cancer patient data including 202 RNA-seq profiles (TCGA database), 315 microarray profiles (GSE2109), and 10 proteome profiles (ProteomeXchange database). We integrated each omics data profile into Recon3D by each individual integration approach, leading to a total of 3740 CGEMs (Figure 2). 

### 3.2. Evaluation of the Quality of a Context-Specific Model of Cancer

MEMOTE is a standardized means of quality control for newly reconstructed GEMs [45]. However, the most effective way for testing the quality of context-specific models is assessing the model functionalities. There are a limited number of ways in which the functionalities of GEMs can be tested, e.g., prediction of the growth rate [46]. Due to the lack of an accurate human objective function [47] and sufficient experimental data to be compared with CGEM results, the objective function-related functionalities of GEMs were not included in this study. Therefore, we made specific indices consisting of metabolic and gene set hallmarks of cancers to evaluate the quality of CGEMs.

#### 3.2.1. Assessment of Metabolic Hallmarks of Cancer in all CGEMs

We started our study with the Warburg effect phenotype as a prominent hallmark of all cancers. We used two specific indices of metabolic hallmarks of cancer to quantify the Warburg effect: the ratio of glycolytic to oxidative ATP flux (i.e., AFR score) and the ratio of extracellular acidification rate (ECAR, a proxy of lactate secretion) to the oxygen consumption rate (i.e., EOR score). Both scores correlate significantly with migration rates, however, AFR has more predictive power than the EOR score [20]. Our results show that all CGEMs have a positive value for AFR and negative for EOR that are in a good agreement with the metabolic behavior of any cancer [35]. Conceivably, the CGEMs generated with the iMAT algorithm are not able to accurately predict the EOR score for the NCI-60 proteome cell line and all patients’ omics data (Table 3). Although AFR and EOR scores have various values in different CGEMs, we noticed that higher AFR values denote more Warburg phenotype cancer cells [20]. Interestingly, it has been recently proposed that ATP plays a major role in the fitness of Warburg cells in their variable microenvironment [48].

#### 3.2.2. Assessment of a Gene Set Hallmark of Cancer in all CGEMs

**Figure 3:**
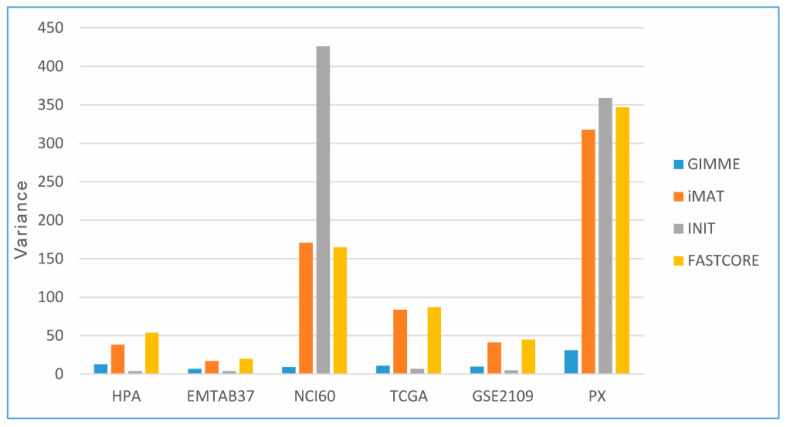
The variance distribution of activated cancer genes among CGEMs. Proteome-based CGEMs show the highest variance distribution of activated genes.

Then, we used a gene set hallmark of cancer to quantify and validate the cancerous molecular signature of CGEMs. This gene set consists of a collection of 197 genes as cancer biomarkers [36]. Among the whole set, 192 genes initially existed in Recon3D. Therefore, we tested how likely this gene set is to be actuated in the generated CGEMs. Table 4 summarizes the percentage of activated gene signatures in different constructed CGEMs. Almost all models have nearly 80–90% of activated cancer genes, however, iMAT- and FASTCORE-based CGEMs poorly (less than 80%) predicted this determined index. On the other hand, GIMME- and INIT-based CGEMs contain the largest amount (93%) of the activated cancer gene set (regardless of the integrated omics data). In addition, proteome-based CGEMs (both cell lines and patient samples) show the highest variance of activated genes, which means the data points are spread out from the mean and from one another (Figure 3).

### 3.3. Evaluation of the Impact of Integration Algorithms and Omics Data on the Behavior of CGEMs

The four integration algorithms differ in assumptions and mathematical formulations in using different gene expression datasets (in the form of microarray, RNA-seq, and proteome data). The iMAT algorithm splits gene expression values into two groups: highly and poorly expressed genes. It then maximizes the model consistency with the classification. Similar to iMAT, the FASTCORE algorithm takes a core set of active reactions in the context of interest as an input. In a different fashion, the GIMME algorithm minimizes the utilization of inactive reactions, and INIT uses the relaxation strategy to prevent the removal of reactions that have a small net accumulation rate. Regarding all the facts mentioned about integration algorithms, we expected that GIMME and INIT would return CGEMs with more reactions and genes including the signature of cancer phenotype. Additionally, our results indicated that GIMME and INIT generated more consistent CGEMs (with less variance in all three scores) (Table 3 and Table 4).

In principle, we expected that proteome data should build more accurate CGEMs than transcriptome data. However, we found no advantages of using proteomic data over transcriptomic data, probably due to the smaller number of expressed genes detected by proteome-based approaches. In contrast, microarray- and RNA-seq-generated CGEMs showed no difference. On the other hand, proteome-based CGEMs (both cell lines and patient samples) were less consistent (with high variance in all three scores) (Table 3 and Table 4).

### 3.4. Characterization of Metabolic Pattern (FBA-Based Feature) in CGEMs

In addition to the evaluation of cancer hallmarks (Warburg scores and gene set hallmarks) in CGEMs, we assessed the similarity of the metabolic patterns among CGEMs to be able to understand the metabolic pattern that makes cancers of widely different types unique. For this reason, we considered two aspects of a metabolic pattern: the active reactions (carrying flux) and the corresponding flux magnitude. The similarity of different types of cancer was investigated by calculating the number of active reactions (carrying flux reactions) using the Jaccard index, and by comparing the flux states (flux magnitude of active reactions that are obtained by FBA) using Bland–Altman plots that will be elaborated on in the next sections.

#### 3.4.1. Number of Active Reactions (Carrying Flux Reactions)

**Figure 4:**
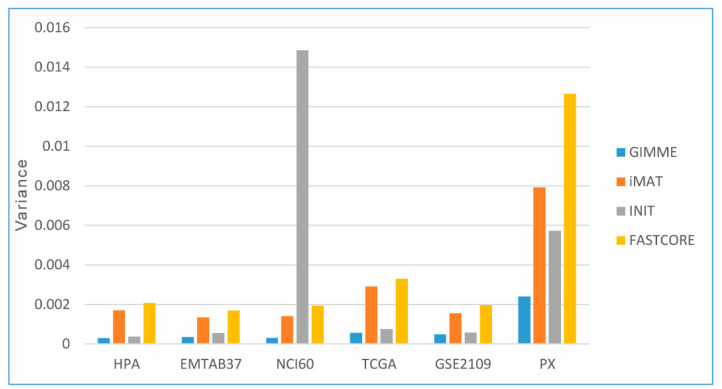
The Jaccard index variance distribution in all CGEMs. Proteome-based CGEMs show the highest variance distribution of active reactions.

The mean value of the Jaccard similarity index indicates the similarities among the number of active reactions in the metabolic subsystems (Recon3D consists of 103 metabolic subsystems) of CGEMs, as presented in Table 5. Figure 4 also represents the Jaccard index variance distribution in all CGEMs. As we expected, GIMME and INIT show less variance in the Jaccard similarity (models are more consistent), in contrast to the iMAT and FASTCORE algorithms. That means, however, that the number of active reactions is highly correlated with the applied integration method. From another point of view, the number of active reactions correlated with the type of input data, meaning that proteome-based CGEMs (both cell lines and patient samples) show the highest variance in our study. Broadly speaking, the number of active reactions might be an appropriate indicator to distinguish CGEMs or evaluate an integration approach.

#### 3.4.2. Flux States of CGEMs (Magnitude Flux of Active Reactions)

In addition to the correlation of a number of active reactions between CGEMs, we also evaluated another aspect of CGEMs, flux states (FBA-based feature). In other words, we took into account the combination of the active reaction and their associated magnitude. Therefore, we leveraged the differences in metabolic flux states among all CGEMs in each dataset–algorithm category to identify the main metabolic features of CGEMs.

### 3.5. Flux States Are an Appropriate Index to Distinguish CGEMs

In this section, we show the Bland–Altman approach used to find that flux states of CGEMs are in agreement. A Bland–Altman plot quantifies the agreement between two quantitative measurements by constructing the limits of agreement [49]. By using the mean and standard deviation (SD) of the differences between two measurements, we calculated statistical limits. Usually, the outcome is a scatter plot in which the difference of the two paired measurements is plotted versus the mean of the two measurements. It was suggested that 95% of the data points should lie within ±2SD of the mean difference.

**Figure 5:**
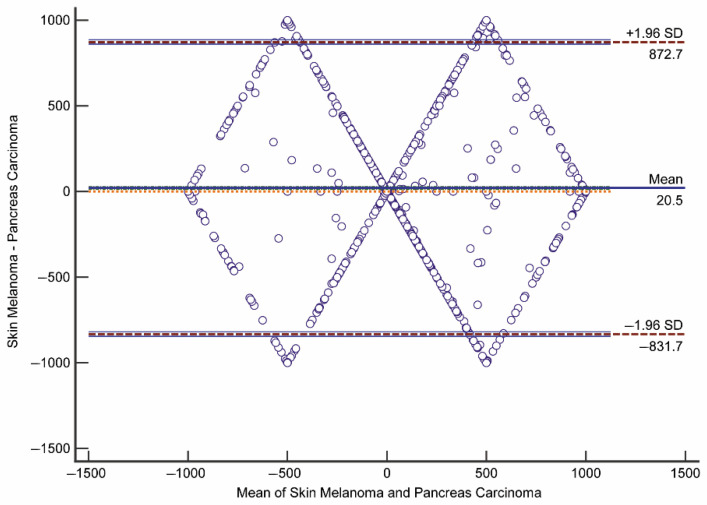
The Bland–Altman plot for flux states of skin melanoma GEM and pancreas carcinoma GEM in EMTAB-37–GIMME method (hypothesis mean = 0 rejected by *p*-value < 0.0001).

Alternatively, it is also possible to plot the differences as percentages [50]. By using the Bland–Altman plot, we answered the following questions: (i) is the discrepancy between two CGEMs large enough to be important?; (ii) how wide are the limits of agreement? If the limits are narrow, then the two flux states of CGEMs are essentially equivalent. Thus, we did pairwise comparisons between any two CGEMs in each dataset–algorithm category and among different datasets to investigate whether each CGEM has a distinct flux fingerprint or CGEMs have the same flux pattern (i.e., two CGEMs are in agreement). The Bland–Altman results show that the flux distributions of any two CGEMs are in disagreement (Table 6). In one example (Figure 5), we depict the plot of differences in flux distribution between skin melanoma and pancreas carcinoma (constructed by EMTAB-37–GIMME), expressed as percentages of the values on the axis ((skin melanoma—pancreas carcinoma)/mean%)), versus the mean of the two measurements. The lines of the 95% confidence interval (CI) for the mean of differences and limits of agreement were drawn. Taken together, these results are highly convincing that flux states are also an appropriate index to distinguish CGEMs.

### 3.6. Are CGEMs Distinguishable Based on Their Flux States?

**Figure 6:**
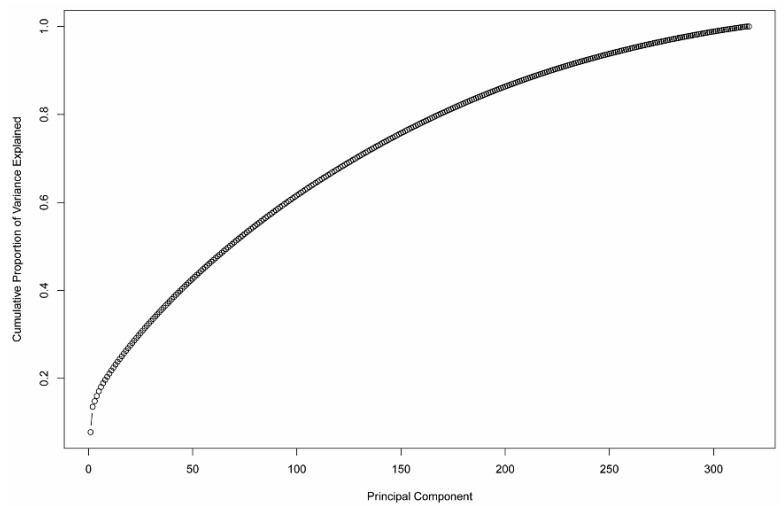
The cumulative variance plot of PCA analysis for EMTAB-37 dataset and GIMME method.

We then used principal component analysis (PCA) to make uncorrelated variables concise and identify similarity amongst clusters in different samples. We could not find a clear separation among CGEMs generated based on proteome data (regardless of the integration methods) and CGEMs generated by FASTCORE (regardless of the omics data). Table 7 shows the number of clusters determined by the first two PCs in which iMAT-based CGEMs have fewer components than the two other approaches, GIMME and INIT. Our analysis showed that PCA is not able to significantly reduce the predictors (reactions) without compromising on explained variance. For example, as shown in Figure 6, a cumulative variance plot for the HPA dataset using the GIMME method, nearly all 32 components are required to explain the variance in the dataset.

### 3.7. Main FBA-Based Features to Distinguish CGEMs

**Figure 7:**
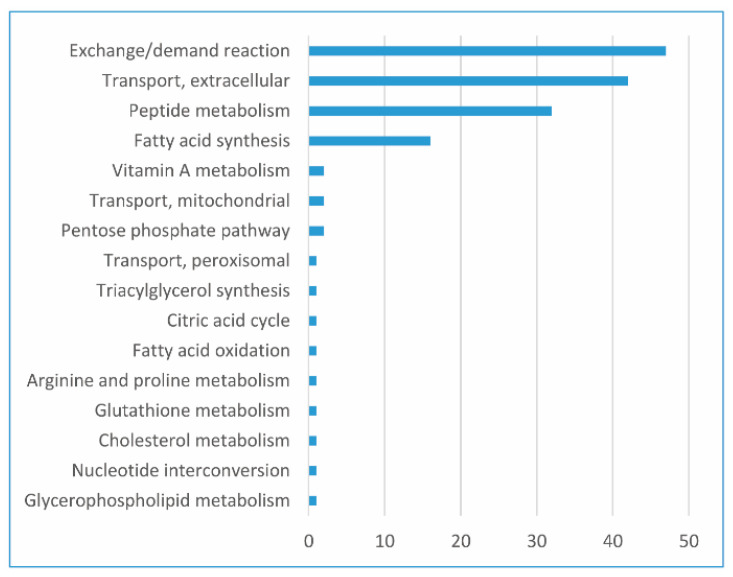
The frequencies of subsystems of important features (*x*-axis, the number of reactions) which were selected in random forest analysis for all omics integration CGEMs.

**Figure 8:**
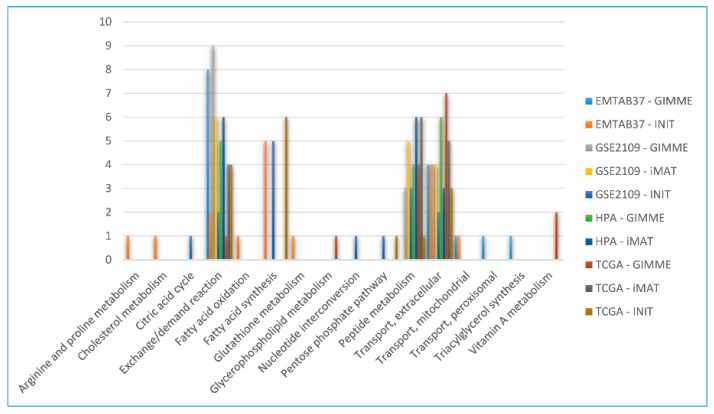
The frequencies of subsystems of important features (reactions) which were selected in the random forest analysis by different dataset–algorithm CGEMs.

Data science provides a plethora of classification algorithms such as logistic regression, support vector machines, naïve Bayes classifiers, decision trees, and the RF classifier [51]. An enhanced version of reaction selection using RF was utilized in order to achieve the best FBA-based feature to distinguish the metabolic pattern in CGEMs. Thus, RF returned a selection of features for each dataset–algorithm CGEM, and we obtained the distinctive features to distinguish CGEMs (we excluded proteome-based and FASTCORE-based CGEMs for the RF analysis). Additionally, RF returned nothing for iMAT–EMTAB-37 and INIT–HPA. Figure 7 represents the subsystems of important feature selection results for all dataset–algorithm CGEMs. In fact, we found different features for each dataset–algorithm (Figure 8). Although different datasets and different integration approaches (Figure 8) selected different flux fingerprints, extracellular transport and peptide metabolism are common features in all CGEMs. In total, in EMTAB-37–GIMME, 15 reactions in five different subsystems (triacylglycerol synthesis, exchange/demand reaction, transport—extracellular, transport—peroxisomal, and transport—mitochondrial), and in EMTAB-37–INIT, 16 reactions in eight different subsystems (exchange/demand reaction, transport—extracellular, transport—mitochondrial, glutathione metabolism, fatty acid synthesis, fatty acid oxidation, arginine and proline metabolism, and cholesterol metabolism), show significant differences in the flux among the clusters. In GSE2109–GIMME, 16 reactions in three different subsystems (exchange/demand reaction, transport—extracellular, and peptide metabolism), in GSE2109–INIT, 15 reactions in seven different subsystems (exchange/demand reaction, fatty acid synthesis, pentose phosphate pathway, transport—extracellular, peptide metabolism, citric acid cycle, and nucleotide interconversion), and in GSE2109–iMAT, 15 reactions in three different subsystems (exchange/demand reaction, peptide metabolism, and transport—extracellular), show significant differences in the flux among the clusters. In HPA–GIMME, 15 reactions in three different subsystems (exchange/demand reaction, peptide metabolism, and transport—extracellular) and in HPA–iMAT, 15 reactions in three different subsystems (exchange/demand reaction, peptide metabolism, and transport—extracellular), show significant differences in the flux among the clusters. In TCGA–GIMME, 15 reactions in five different subsystems (transport—extracellular, exchange/demand reaction, glycerophospholipid metabolism, peptide metabolism, and vitamin A metabolism), in TCGA–INIT, 15 reactions in five different subsystems (transport—extracellular, fatty acid synthesis, exchange/demand reaction, peptide metabolism, and pentose phosphate pathway), and in TCGA–iMAT, 15 reactions in three different subsystems (transport—extracellular, exchange/demand reaction, and peptide metabolism), show significant differences in the flux among the clusters. Interestingly, our results show that the main features (regardless of data source and extraction algorithms) to distinguish CGEMs are extracellular transport, peptide metabolism, fatty acid synthesis, vitamin A metabolism, mitochondrial transporters, and the pentose phosphate pathway.

### 3.8. Development of the GEMbench Interactive Website

**Figure 9:**
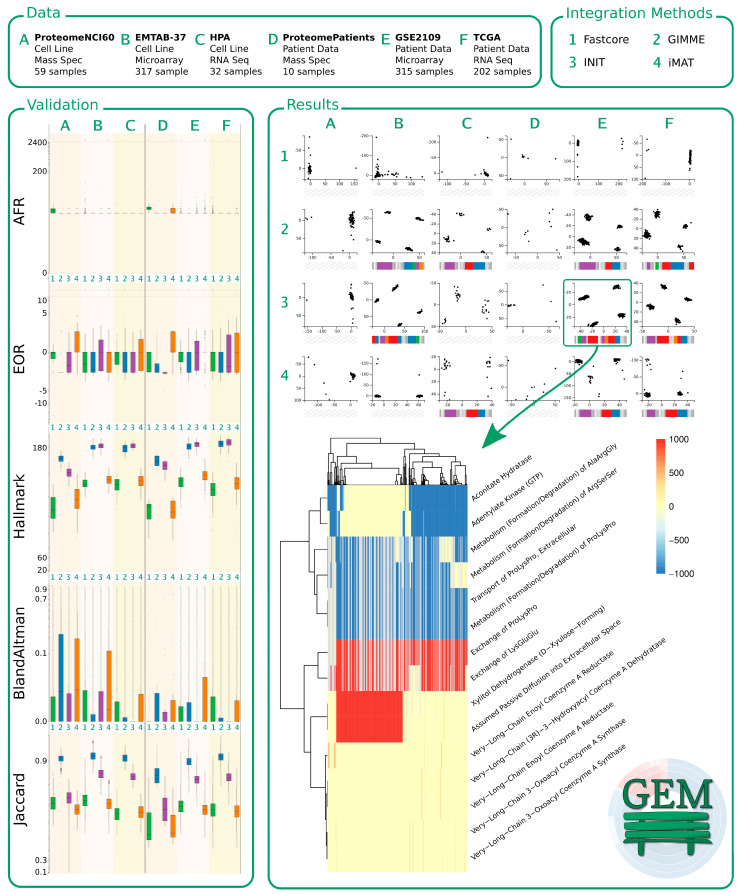
Preview of the GEMbench website. The top of the figure shows some basic information regarding the data (A: NCI-60 Proteome, B: EMTAB-37, C: HPA, D: ProteomePatients, E: GSE2109, and F: TCGA) and integration methods (1: FASTCORE, 2: GIMME, 3: INIT, and 4: iMAT) used in this study. The panel on the left-hand side shows the results of validating our CGEMs. The panel on the right-hand side shows some of the results: at the top can be seen how we distinguished groups of samples using PCA and at the bottom, a corresponding heatmap indicates the results when analyzing the data of 3E (INIT applied to GSE2019) using random forests.

We believe that static figures do not give sufficient access to the data. Thus, we built a website that allows users to explore our findings using interactive visualizations. Apart from the landing page, the website consists of three main pages describing (i) the data we obtained, (ii) the integration methods we used, and (iii) the findings we observed, and parts of it are depicted in Figure 9. On the findings page of the website, our validation results can be comprehended interactively. We dynamically visualized the data using boxplots according to the five different metrics, as explained above (top to bottom: AFR, EOR, hallmark, Bland–Altman, and Jaccard index). The boxplots are indicated on the left-hand side of Figure 9. The non-linear scales of the y-axes in the figure on the webpage are dynamically scaled to zoom into dense areas of high or low values (we removed most tick marks in Figure 9 and increased the font size of the remaining labels to improve readability).

Every plot contains six groups of boxplots (representing the underlying data A–F, as indicated by the slightly different background colors), every group consist of four boxplots representing the applied integration methods (1–4: green, blue, purple, and orange).

On the website, the boxes and the outliers can be hovered over to get more information about the data, or a boxplot can be clicked to explore the outliers in a modal window. Our results of the cluster analyses can be explored when selecting the clusterability metric on the findings page, indicated on the right-hand side of Figure 9. It initially displays our PCA results of the different integration methods (vertically 1–4) and data samples (horizontally A–F). Every scatter plot categorizes the corresponding data/method samples according to the first two principal components. If we were able to distinguish clusters, we ran an RF analysis and analyzed which cellular processes contributed to the differentiation of the samples (as shown in the heatmap at the bottom of Figure 9).

Based on the results of the RF, we determined which cellular subsystems were affected by the corresponding processes and found 16 subsystems to be major targets of changes. Based on that insight, we developed colored barcodes as shown below each PCA plot. Every barcode represents a cellular subsystem. If a barcode is colored, a process of the corresponding subsystem shows a different expression between the clusters of data. The bigger the colored section, the more processes of the subsystem are affected. As not all data/method combinations expressed proper clusters, we were not able to identify differentially expressed processes and, thus, not all PCA plots have a barcode. On the website, the barcodes are clickable and reveal more details of the RF results.

### 3.9. Limitations

Drawing conclusions from metabolic heterogeneity in different types of cancers and identifying cancer-specific metabolic signatures should be a much more precise and high-resolution process than finding FBA-based features using PCA and RF. This prediction is not entirely definitive to find metabolic signatures of cancers. Complementary and supplementary approaches can reinforce the results.

## 4. Discussion

Cancer is one of the most complicated diseases with multiple aggressive phenotypes and shows heterogeneity and diversity in the underlying (epi)genetic alteration, gene expression, and metabolic pattern. In spite of many systematic investigations of the genome, epigenome, and transcriptome patterns of the cancerization process [52], the metabolome profile of all cancers (similarity and differences amongst all types of cancer) has not been fully studied and there has not been a comprehensive study to systematically assess the metabolic pattern of the cancerization process from the early stage to metastasis of disease.

In our current study, we employed ML approaches to study the theoretical landscape of cancer metabolism, i.e., to determine the best FBA-based feature for cancer stratification. Additionally, we benchmarked context-specific models of cancer metabolism, which can let researchers to choose the proper integration methods and omics data for generating CGEMs.

In a first step, we evaluated the influence of different omics data and extraction algorithms in the development of cancer models. By measuring and analyzing two Warburg scores (AFR and EOR) and a cancer gene dataset of all 3740 generated CGEMs, we demonstrated that all approaches (regardless of platform and extraction methods) are able to generate a CGEM. Amongst the omics data, proteome-based CGEM measurements, and amongst the algorithms, iMAT and FASTCORE, showed less accuracy in CGEMs. The lower accuracy of proteome data may be due to the smaller number of expressed genes detected by proteomic-based approaches.

There are several hypotheses about the metabolic hallmarks of cancers. In this study, we specifically investigated whether the similar metabolic pattern represents these hallmarks of cancers with a focus on the Warburg effect as an example of these metabolic phenotypes.

In a second step, we were further motivated to discover the metabolic patterns in CGEMs and highlight the FBA-based features that might distinguish the CGEMs. These analyses lead us to a critical view of cancer metabolism, which may pave the way for personalized cancer treatment. In fact, assessment of heterogeneity in the metabolic pattern of cancers underlines the requirement for personalized medicine for cancer treatment.

Accordingly, we assessed the metabolic pattern from two perspectives, i.e., the number of activated reactions and the FBA-based features of CGEMs. Our results show that the number of activated reactions might be a suitable feature for CGEM comparison and evaluation. On the other hand, the flux states of CGEMs are also an appropriate alternative framework to distinguish CGEMs.

Although, according to the central dogma that different biological layers should be correlated with each other (level of transcripts and proteins with enzyme activities), numerous published studies did not confirm this fact [53]. Nonetheless, the flux states of a biological system as a last informative layer of the central dogma (that is a representative of enzyme activity) could be the most informative layer. Consequently, by running FBA, we obtained the flux distribution of cancer models and, by Bland–Altman analyses, we found that the flux states of each cancer model are like a metabolic fingerprint of the cancer that cannot be used interchangeably. Afterwards, we sought the proper method to cluster the FBA-based features and utilized PCA-based dimensionality reduction and random forest approaches to reveal the most influential flux containing the most relevant information.

Various experimental studies investigated the heterogeneity of metabolic pattern and suggested several mechanisms for the underlying metabolic alterations across cancers. In one of the most recent studies, Reznik et al. [4] suggested the recurrent patterns of differentially abundant metabolites across cancer types. In another investigation, it has been demonstrated that disturbance in vitamin A metabolism [54] and mitochondrial dysfunction [55] likewise seem to be important characteristics of cancers.

Interestingly, we discovered that extracellular transport, peptide metabolism, fatty acid synthesis, vitamin A metabolism, mitochondrial transporters, and the pentose phosphate pathway are probably the main candidates for cancer heterogeneity and stratification, which is in good agreement with previous findings. In fact, these pathways and subsystems not only enable us to distinguish between cancer cells and normal counterparts, but also provide the different metabolic patterns in cancers.

Conclusively, this study suggests that GIMME and INIT algorithms as well as microarray and RNA-seq datasets might be better suited dataset–algorithm combinations for the reconstruction of context-specific models of cancers. Moreover, the Warburg effect is the consequence of different metabolic patterns of cancers. We believe that these results have the potential to further establish a new discovery approach in cancer metabolism and personalized medicine and may address an unmet need for precision treatments of cancer patients.

## 5. Conclusions

Altogether, this study suggested that the differences in the flux profiles of cancers is nearly unique and could act as a cancer metabolic fingerprint. Indeed, the heterogeneity in the metabolic pattern for cancers contributes to better cancer stratification and personalized treatment.

## Figures and Tables

**Table 1:** Summary of studies that used omics databases and integration algorithms to generate CGEM.

Reference	Study	Conclusions
Shlomi et al. [16]	Generation of cancer-specific GEM (CGEM) by omics data integration to investigate the metabolic reprogramming of 60 cell lines by employing stoichiometric and enzyme solvent capacity constraints.	Warburg effect is a direct consequence of the metabolic adaptation to maximize the growth rate of cancer cells.
Folger et al. [17]	Combination of microarray data from 59 cancer cell lines with Recon1 to generate a generic metabolic model of human cancer cells.	Generation of a generic metabolic model of human cancer cells.
Wang et al. [18]	Reconstruction and pathway-level analyses of GEMs for 26 human cancer and counterpart normal tissues.	Eicosanoid metabolic pathway is a potential selective drug target.
Jerby et al. [19]	Development of a method to interpret metabolic phenotypes of breast cancer patients by the integration of transcriptomics and proteomics data into the GEMs.	Presentation of a novel metabolic hallmark of estrogen receptor in breast cancer.
Yizhak et al. [20]	Exploration of the role of the Warburg effect in supporting tumor migration by using GEMs.	Identification of metabolic targets inhibiting the cancer cell migration.
Nam et al. [21]	Deployment of genetic mutation information, transcriptomics data of nine cancer types as well as Recon2 for the reconstruction of CGEMs.	Prediction of oncometabolites that potentially dysregulate the epigenetic mechanisms.
Asgari et al. [22]	Construction of 13 CGEMs and their corresponding normal models by gene expression integration into Recon1 using the E-Flux method.	Results supporting the hypothesis that the Warburg effect is a consequence of metabolic adaptation.
Uhlen et al. [23]	Generation of more than 7000 patient-specific GEMs based on the integration of transcriptomics data into HMR2 by the tINIT method.	They disclosed that cancer patients show widespread metabolic heterogeneity, highlighting the need for personalized treatment.

**Table 2:** The sources, platforms, and the number of samples we used in this study.

Sources	Platforms	Samples
HPA	RNA-seq	32 cell lines
EMTAB-37	Microarray	317 cell lines
NCI-60	Mass spectrometry proteomics	59 cell lines
TCGA	RNA-seq	202 patients
GSE2019	Microarray	315 patients
PX	Mass spectrometry proteomics	10 patients

**Table 3:** The mean of AFR and EOR scores in all CGEMs. AFR and EOR are two indices to quantify Warburg effect in CGEMs.

	GIMME		iMAT		INIT		FASTCORE	
	AFR	EOR	AFR	EOR	AFR	EOR	AFR	EOR
HPA	1.00	−0.31	1.03	−0.05	1.00	−0.44	1.32	−0.14
EMTAB-37	1.00	−0.44	1.11	−0.31	1.17	−0.06	1.62	−0.52
NCI−60	1.00	−0.76	1.10	0.63	1.07	−0.41	1.65	−0.50
TCGA	1.00	−0.38	1.12	0.00	1.00	−0.22	17.40	−0.28
GSE2109	1.01	−0.48	1.33	0.06	1.20	−0.10	1.27	−0.21
PX	1.00	−0.55	2.54	0.18	1.08	−1.17	4.66	−0.39

**Table 4:** The percentage of activated cancer genes in CGEMs of different datasets and methods.

	GIMME	iMAT	INIT	FASTCORE
HPA	91.15	77.93	92.26	75.98
EMTAB-37	91.39	78.67	92.12	76.84
NCI-60	87.20	69.61	79.66	64.96
TCGA	92.69	76.84	93.11	73.85
GSE2109	91.90	80.31	92.55	77.38
PX	86.14	62.18	80.76	61.52

**Table 5:** The mean value of Jaccard similarity index among the number of active reactions in the metabolic subsystems.

Dataset–Algorithm	GIMME						iMAT						INIT						FASTCORE					
	HPA	EMTAB-37	NCI-60	TCGA	GSE2019	PX	HPA	EMTAB-37	NCI-60	TCGA	GSE2019	PX	HPA	EMTAB-37	NCI-60	TCGA	GSE2019	PX	HPA	EMTAB-37	NCI-60	TCGA	GSE2019	PX
Mean value	0.91301	0.91918	0.90782	0.91436	0.89615	0.84622	0.65933	0.70365	0.67382	0.66188	0.67305	0.57403	0.83354	0.84521	0.71024	0.82953	0.82020	0.68047	0.65023	0.72104	0.70777	0.67031	0.69142	0.56654

**Table 6:** The percentage of statistically significant disagreement among CGEM pairs in Bland–Altman analysis (*p*-value < 0.05).

	GIMME	iMAT	INIT	FASTCORE
HPA	88.71	82.46	88.91	83.87
EMTAB-37	87.10	69.72	81.48	81.69
NCI-60	61.31	62.71	82.29	84.98
TCGA	88.44	83.65	90.27	83.01
GSE2109	84.05	77.91	89.16	85.54
PX	80.00	86.67	82.22	93.33

**Table 7:** The number of clusters, which were determined by the first two principal components.

	GIMME	iMAT	INIT	FASTCORE
HPA	4	2	-	1
EMTAB-37	4	2	4	1
NCI-60	1	1	1	1
TCGA	4	2	4	1
GSE2109	4	2	4	1
PX	-	-	1	1

## Data Availability

The results presented in this study are openly available at https://gembench.bio.informatik.uni-rostock.de/.
